# Decreased prothrombin conversion and reduced thrombin inactivation explain rebalanced thrombin generation in liver cirrhosis

**DOI:** 10.1371/journal.pone.0177020

**Published:** 2017-05-04

**Authors:** Romy M. W. Kremers, Marie-Claire Kleinegris, Marisa Ninivaggi, Bas de Laat, Hugo ten Cate, Ger H. Koek, Rob J. Wagenvoord, H. Coenraad Hemker

**Affiliations:** 1 Synapse Research Institute, Cardiovascular Research Institute Maastricht, Maastricht University, Maastricht, the Netherlands; 2 Department of Biochemistry, Cardiovascular Research Institute Maastricht, Maastricht University, Maastricht, the Netherlands; 3 Department of Internal Medicine, Division of Gastroenterology and Hepatology, Maastricht University Medical Center, Maastricht, the Netherlands; University Medical Center, Freiburg, GERMANY

## Abstract

Impaired coagulation factor synthesis in cirrhosis causes a reduction of most pro- and anticoagulant factors. Cirrhosis patients show no clear bleeding or thrombotic phenotype, although they are at risk for both types of hemostatic event. Thrombin generation (TG) is a global coagulation test and its outcome depends on underlying pro- and anticoagulant processes (prothrombin conversion and thrombin inactivation). We quantified the prothrombin conversion and thrombin inactivation during TG in 30 healthy subjects and 52 Child-Pugh (CP-) A, 15 CP-B and 6 CP-C cirrhosis patients to test the hypothesis that coagulation is rebalanced in liver cirrhosis patients. Both prothrombin conversion and thrombin inactivation are reduced in cirrhosis patients. The effect on pro- and anticoagulant processes partially cancel each other out and as a result TG is comparable at 5 pM tissue factor between healthy subjects and patients. This supports the hypothesis of rebalanced hemostasis, as TG in cirrhosis patients remains within the normal range, despite large changes in prothrombin conversion and thrombin inactivation. Nevertheless, *in silico* analysis shows that normalization of either prothrombin conversion or thrombin inactivation to physiological levels, by for example the administration of prothrombin complex concentrates would cause an elevation of TG, whereas the normalization of both simultaneously maintains a balanced TG. Therefore, cirrhosis patients might require adapted hemostatic treatment.

## Introduction

Liver cirrhosis causes disturbances of blood coagulation and alterations of platelet function and number [1]. Plasma levels of both procoagulant (FII, FV, FVII, FIX, FX, and FXI) and the anticoagulant factors (protein C, protein S, and antithrombin) are reduced due to diminished production by the liver [2–5]. Although coagulation factor levels in liver cirrhosis patients can be as low as the levels found in congenital deficiency, the symptoms associated with deficiency are absent in cirrhosis patients [2–5]. The most commonly reported hemostatic problems in liver cirrhosis are bleeding from ruptured esophageal varices, bruising, bleeding after invasive procedure, but also deep venous thrombosis, pulmonary embolism and intrahepatic thrombus formation [6–11]. Although both bleeding and thrombosis have been reported in cirrhosis patients, routine clinical test such as the prothrombin time (PT) and the activated partial thromboplastin time (aPTT) indicate an increased bleeding risk [3,12]. The PT is often prolonged because of a reduction of procoagulant factor levels and if the PT is modified to include the effect of anticoagulant factors, no difference is found between liver cirrhosis patients and healthy subjects [1,12,13]. During the last decade, a new view of coagulation in liver cirrhosis patients emerged: the rebalanced coagulation system [1]. It was previously hypothesized that a reduction of both the pro- and anticoagulant pathways results in a newly found balance in coagulation [1,6,14] and this paper lends quantitative support to this concept.

Routine coagulation tests do not correlate with the bleeding nor thrombotic risk in liver cirrhosis, mainly because they only reflect part of the coagulation system. The thrombin generation test (TG), which represents the complete system, correlates better with the hemostatic situation described in cirrhosis patients [2,4,5,12,15,16]. Thrombin generation in plasma is dependent on two underlying processes, the production of thrombin (i.e. prothrombin conversion) and the removal of thrombin from the clotting plasma (thrombin inactivation) [17]. Prothrombin conversion is affected by the levels of all procoagulant factors, but also by the levels of proteins C and S. The latter factors inactivate FVa and FVIIIa and thereby decrease prothrombin conversion [18]. The major players in thrombin inactivation are antithrombin (AT) and α_2_-macroglobulin (α_2_M) [19].

We have recently developed a method to determine the prothrombin conversion and thrombin inactivation curves from a TG curve by an approach based on computational modeling of thrombin inactivation [20–22]. In this way we can investigate prothrombin conversion and thrombin inactivation separately. In this study we investigated the changes in prothrombin conversion and thrombin inactivation in liver cirrhosis. Additionally, we used computational modeling to investigate the individual contribution of changes in prothrombin conversion and thrombin inactivation to the differences in TG, and to study the consequences of these changes in pro- and anticoagulant processes for the bleeding management in cirrhosis patients.

## Materials and methods

### Sample collection and handling

The population tested in this paper is the same as described by Kleinegris et al [16]. The study was approved by the local medical ethics committee of the Maastricht University Medical Center and healthy volunteers and patients were enrolled in the study after written informed consent, according to the Helsinki declaration. All-cause liver cirrhosis patients were enrolled after diagnosis based on clinical, laboratory, ultrasound, gastroscopy and/or histological evidence. They were classified as Child-Pugh A (n = 52), B (n = 15), and C (n = 6). Exclusion criteria were the use of medication that affects coagulation (vitamin K antagonists, direct thrombin or FXa inhibitors, heparin), documented congenital coagulation disorders and age below 18 years. Blood was collected on 3.2% citrate in a 9:1 ratio for the preparation of platelet poor plasma. Platelet poor plasma was prepared by centrifuging twice at 2821 ∙ g for 10 minutes and was stored at -80°C until further use.

### Materials

Chromogenic thrombin substrate S2238 was synthesized in house. Unfractionated heparin and bovine serum albumin were purchased at Sigma-Aldrich (Zwijndrecht, the Netherlands). Bovine thrombin was purified in house as described by Church [23] and bovine antithrombin according to the protocol of Thaler [24]. Staphylocoagulase was purified in house as described by Hendrix et al [25]. Reagents for thrombin generation were purchased from Thrombinoscope bv (Maastricht, the Netherlands) and recombinant soluble thrombomodulin was a gift from Thrombinoscope bv.

### Measurement of coagulation factor levels

Functional AT, α_2_M and prothrombin levels were measured as previously described [20]. Plasma fibrinogen levels were measured by the Clauss method [26].

### Thrombin generation

Calibrated Automated Thrombinography (CAT) was performed as previously described [17]. All wells contained 80 μl plasma and 20 μl of PPP reagent LOW (1 pM TF, 4 μM phospholipids) or PPP reagent (5 pM TF, 4 μM phospholipids). To calibrator wells, 20 μl of calibrator was added instead. Thrombin generation (TG) was initiated by the addition of 20 μl of ZGGR-AMC (417 μM) and CaCl_2_ (16.7 mM). Recombinant soluble thrombomodulin (0.56 nM f.c.) was added to PPP reagent LOW to test for APC resistance. The TG fluorescence data was converted to thrombin generation curves, as described elsewhere [27] and used to perform additional computational analysis to extract prothrombin conversion curves.

### Calculation of prothrombin conversion and thrombin inactivation

The thrombin generation curve is the net result of prothrombin conversion and thrombin inactivation, and therefore the course of prothrombin conversion can be calculated if thrombin generation and thrombin inactivation are known. Thrombin inactivation was predicted by the previously described and validated computational model based on the plasma antithrombin, α_2_-macroglobulin and fibrinogen level, and was used to determine prothrombin conversion curves from thrombin generation data [20,28].

### In silico experimentation

Thrombin generation is the net result of the underlying processes of prothrombin conversion and thrombin inactivation. Therefore, TG can be calculated if the courses of prothrombin conversion and thrombin inactivation are known. Thrombin inactivation was predicted for each sample by a computational model as described previously [20], and prothrombin conversion curves were obtained from the original experimental data. This methodology makes it not only possible to predict a TG curve, but also to perform *in silico* experimentation to determine the effect of changes in prothrombin conversion or thrombin inactivation in cirrhosis patients.

Prothrombin conversion and/or thrombin inactivation were normalized to average healthy values to test the hypothesis that TG is rebalanced in liver cirrhosis, and that normalization of either prothrombin conversion or thrombin inactivation would result in a deviant hemostatic state in cirrhosis patients. The contribution of changes in prothrombin conversion in liver cirrhosis to TG were determined by simulating TG curves based on the average prothrombin conversion curves of healthy controls and the thrombin inactivation system as found in each individual cirrhosis patient. The effect of acquired AT deficiency in cirrhosis was determined by simulating TG curves based on prothrombin conversion curves as found in cirrhosis patients with the average AT level found in healthy controls (2.08 μM). The role of α_2_M levels was investigated in the same manner.

The administration of prothrombin complex concentrate (PCC) with or without antithrombin supplementation was simulated *in silico* by increasing the prothrombin conversion curve (and the AT levels where indicated) to 110%, 120% and 130%, and the effects on TG were analyzed.

### Statistics

The Statistical Package for the Social Sciences (SPSS) was used to determine the statistical significance of the results. The distribution of the data was tested with a Shapiro-Wilk test and the statistical significance of differences between the groups was determined with ANOVA or Kruskal-Wallis analysis accordingly.

## Results

**Fig 1:**
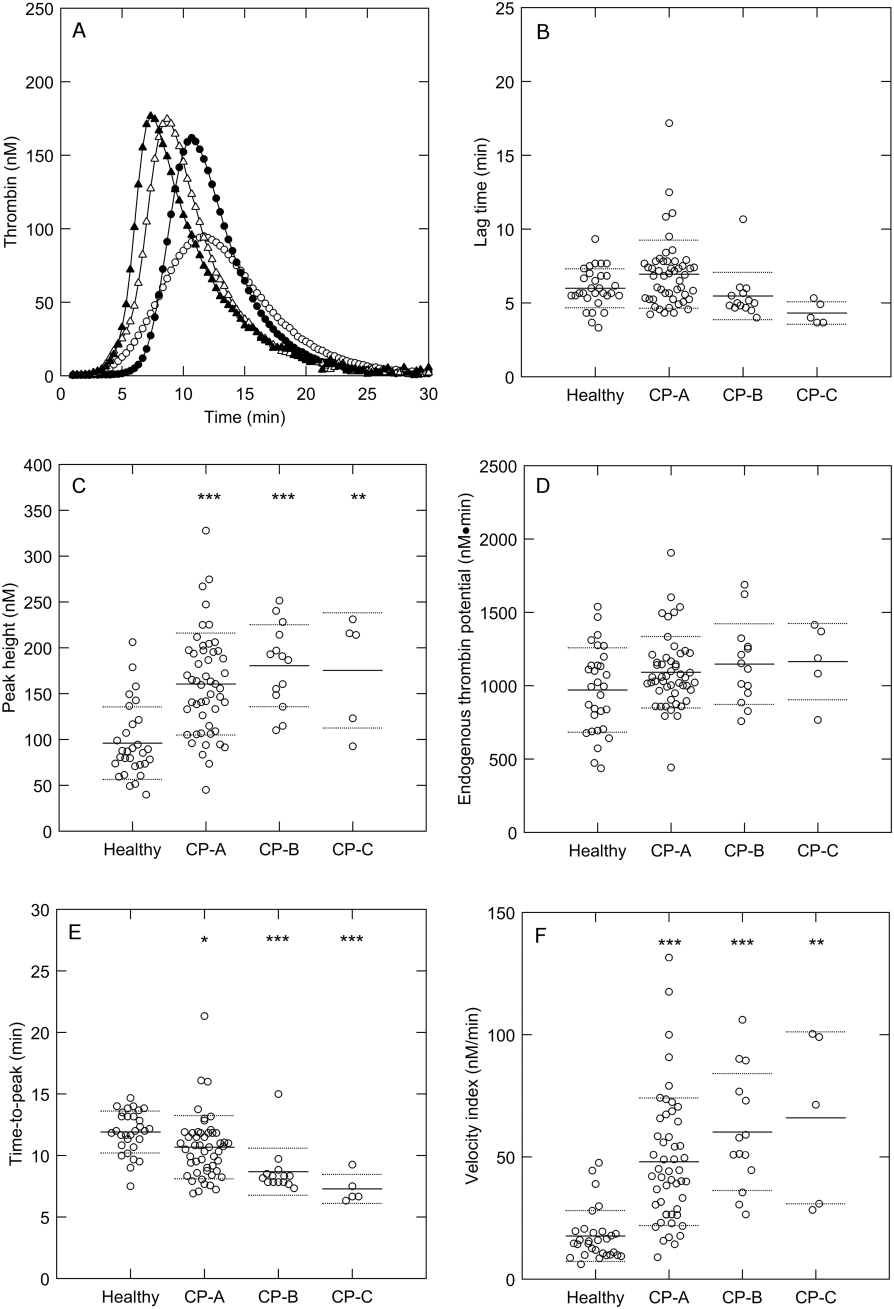
Thrombin generation in cirrhosis patients and healthy subjects. (A) Mean thrombin generation curves in healthy subjects (○), Child-Pugh A patients (●), Child-Pugh B patients (Δ), and Child-Pugh C patients (▲) measured at 1 pM TF. (B) Lag time, (C) peak height, (D) endogenous thrombin potential, (E) time-to-peak and (F) velocity index were quantified from the TG curves. *p<0.05, **p<0.01, ***p<0.001 compared to healthy subject values.

Plasma samples from 30 healthy subjects and 73 liver cirrhosis patients, classified as Child-Pugh (CP)-A, CP-B or CP-C, were collected (Table 1). Thrombin generation was measured at 1 pM TF (Fig 1) and 5 pM TF (S1 Fig). The lag time and endogenous thrombin potential (ETP) were comparable between healthy subjects and cirrhosis patients, whereas the peak height and velocity index were significantly elevated in patients, irrespective of the TF concentration used. Subsequently, the time-to-peak was shorter in patients than in healthy subjects.

**Table 1:** Patient characteristics.

Characteristic	Child-Pugh A (n = 52)	Child-Pugh B (n = 15)	Child-Pugh C (n = 6)
Age (years)	61 (54–67)	53 (51–66)	55 (40–62)
Sex (male, %)	31 (60%)	12 (80%)	5 (83%)
Child-Pugh score	5 (5–5)	8 (7–8)	10 (10–11)
MELD score	7 (7–9)	13 (11–16)	15 (13–18.5)
INR	1.06 (1.02–1.12)	1.21 (1.15–1.30)	1.38 (1.25–1.60)
Platelet count (x 10^9/L)	171 (106–213)	81 (54–103)	102 (66–120)
Cause of cirrhosis			
Alcoholic	26	10	4
Non-alcoholic steatosis hepatitis	4		
Hemochromatosis	4		
Auto-immune hepatitis / Primary biliary cirrhosis	6		
Toxic/medication	3		1
Hepatitis B/C	2	3	
Congenital fibrosis		1	1
Unknown origin	7	1	

Values are expresses as medians with interquartile ranges.

Plasma antithrombin, α_2_-macroglobulin, prothrombin and fibrinogen levels were measured to further characterize the hemostatic state of the subjects and to enable the calculation of prothrombin conversion and thrombin inactivation parameters (Table 2). Plasma AT levels were significantly decreased and some patients had AT levels as low as the levels found in heterozygous AT deficiency (50%). Plasma prothrombin levels were significantly reduced in cirrhosis patients as well. On the contrary, α_2_M levels were significantly increased in CP-A patients. Fibrinogen levels were elevated in CP-A patients, but diminished in CP-C patients.

**Table 2:** Coagulation factor levels.

Coagulation factor	Healthy subjects (n = 30)	Child-Pugh A (n = 52)	Child-Pugh B (n = 15)	Child-Pugh C (n = 6)
Prothrombin, μM (±SD)	1.13 (±0.25)	0.91 (±0.20)***	0.61 (±0.15)***	0.49 (±0.22)***
Antithrombin, μM (±SD)	2.08 (±0.37)	1.65 (±0.57)**	0.99 (±0.49)***	0.81 (±0.59)***
α_2_-macroglobulin, μM (±SD)	3.21 (±0.64)	3.66 (±0.75)*	3.31 (±0.86)	3.17 (±0.69)
Fibrinogen, g/L (±SD)	2.67 (±0.40)	3.27 (±0.79)**	2.83 (±0.92)	1.77 (±0.94)***

*p<0.05,

**p<0.01,

***p<0.001 compared to healthy subject values.

**Fig 2:**
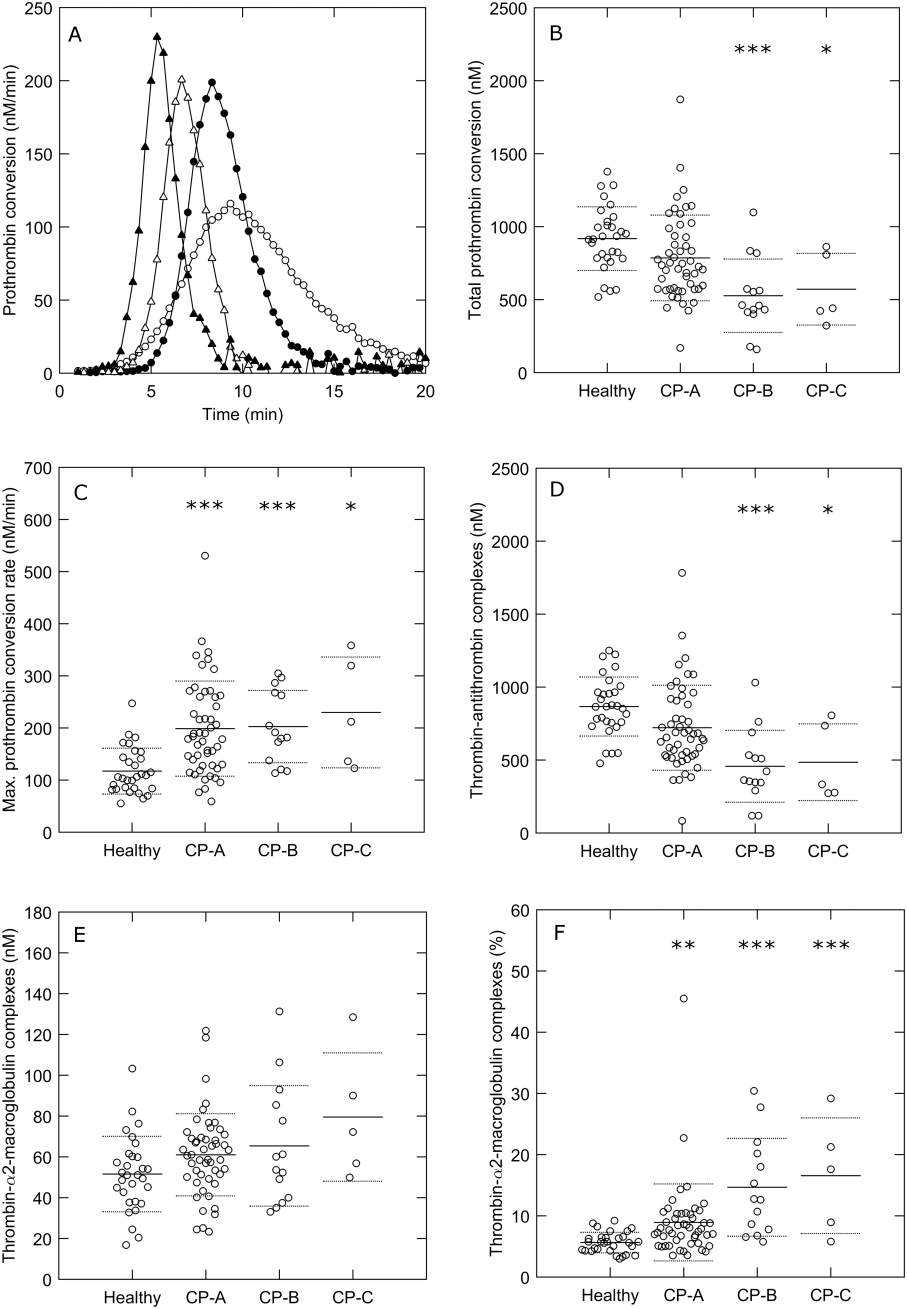
Prothrombin conversion in cirrhosis patients and healthy subjects. (A) Mean prothrombin conversion curves in healthy subjects (○), Child-Pugh A patients (●), Child-Pugh B patients (Δ), and Child-Pugh C patients (▲) triggered with 1 pM TF. (B) Total prothrombin conversion, (C) maximal rate of prothrombin conversion, (D) thrombin-antithrombin formation, (E) thrombin-α_2_-macroglobulin formation and (F) the percentage of thrombin inhibited by α_2_-macroglobulin were quantified from the TG curves. *p<0.05, **p<0.01, ***p<0.001 compared to healthy subject values.

**Fig 3:**
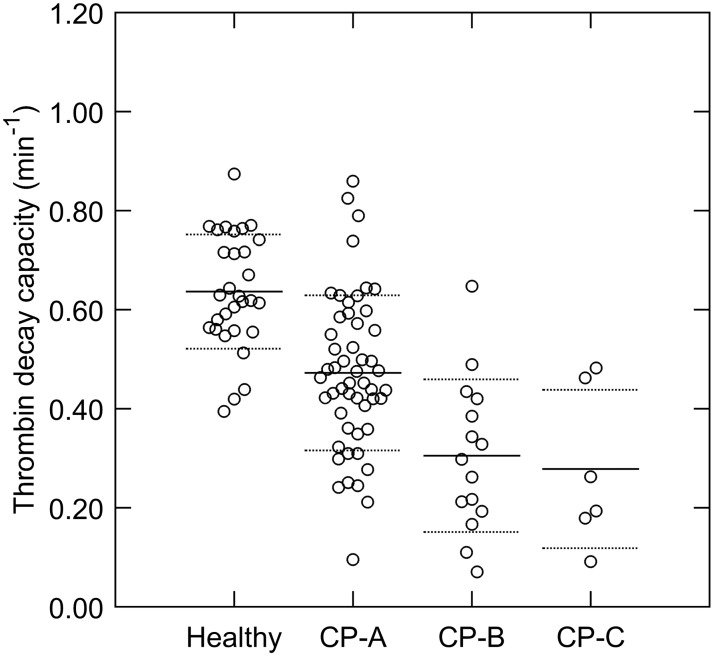
Thrombin inactivation capacity in cirrhosis patients and healthy subjects. The thrombin decay capacity was calculated based on the antithrombin, α_2_-macroglobulin and fibrinogen level of the sample.*p<0.05, **p<0.01, ***p<0.001 compared to healthy subject values.

To study the significance of these differences in coagulation factor levels to the thrombin generation profile, we determined the courses of prothrombin conversion and thrombin inactivation during TG. Fig 2 and S2 Fig show that there is a significant difference in the course of prothrombin conversion between patients and healthy subjects, triggered with 1 and 5 pM TF, respectively. The total amount of prothrombin that is converted during thrombin generation is significantly reduced in cirrhosis patients (15–43%, depending on the disease severity; Fig 2B), which corresponds to the reduced prothrombin plasma levels found in patients (Table 2). The maximal rate of prothrombin conversion (Fig 2C) is higher in patients, indicating that the concentration and/or activity of the prothrombinase complex is increased. In addition to the reduction of prothrombin conversion, we found a marked reduction of thrombin decay capacity in cirrhosis patients (Fig 3). The amount of thrombin-antithrombin complexes that were formed during TG was significantly lower in patients compared to healthy subjects (Fig 2D). On the contrary, thrombin-α_2_-macroglobulin complex formation is comparable in patients and healthy subjects, but the relative amount of thrombin inhibited by α_2_M is elevated in patients (Fig 2E and 2F).

**Fig 4:**
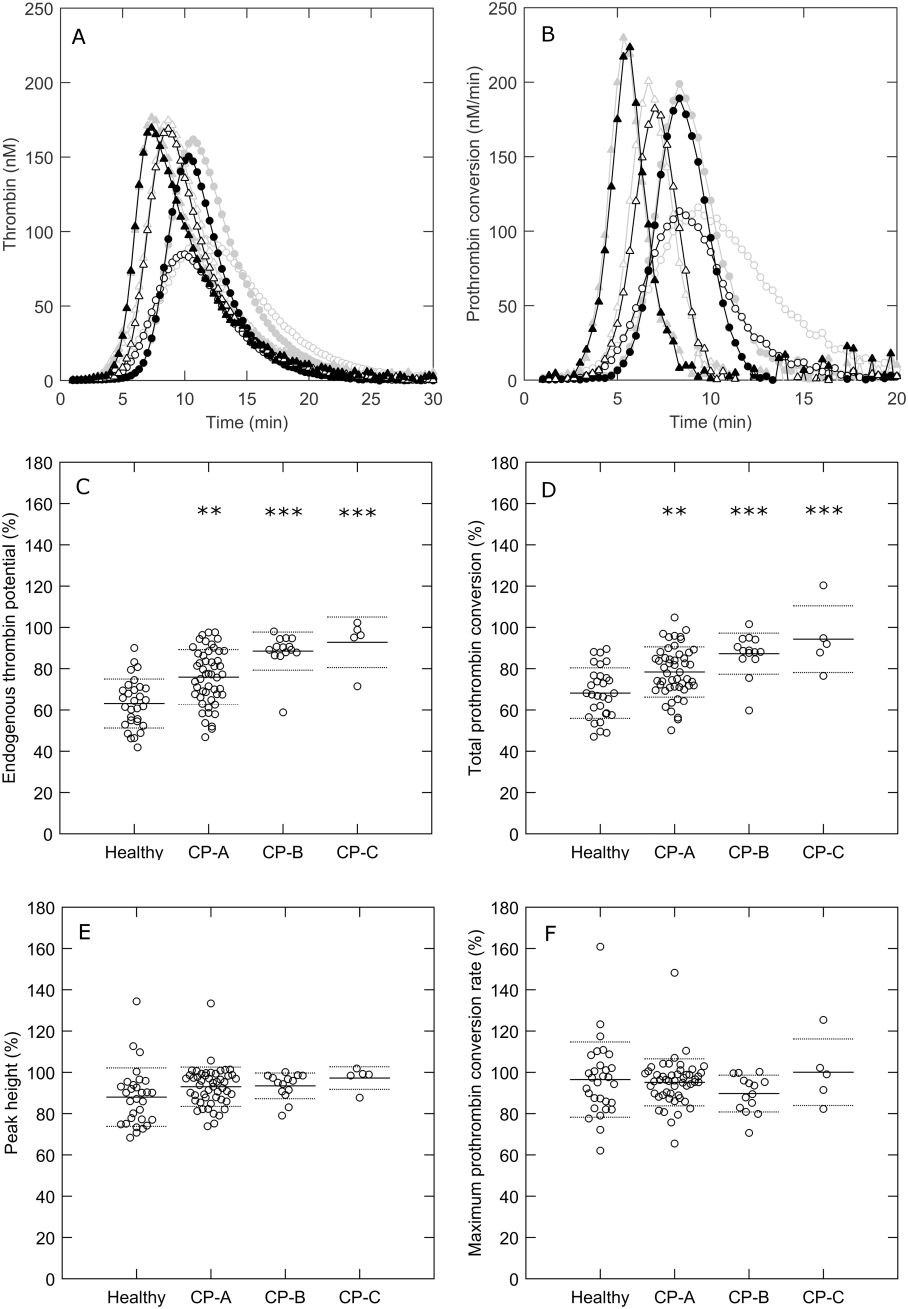
APC sensitivity in cirrhosis patients and healthy subjects. (A-B) Thrombin generation and prothrombin conversion curves measured at 1 pM TF in the absence (gray) or presence of 0.56 nM thrombomodulin (black) in healthy subjects (○), Child-Pugh A patients (●), Child-Pugh B patients (Δ), Child-Pugh C patients (▲). (C) (C-F) The percentage of the ETP, peak height, total prothrombin conversion and the maximum prothrombin conversion rate in plasma with thrombomodulin compared to plasma without thrombomodulin. *p<0.05, **p<0.01, ***p<0.001 compared to healthy subject values.

The function of the APC system was tested by measuring thrombin generation in the presence or absence of 0.56 nM thrombomodulin (Fig 4). In healthy subjects, TM caused a significant reduction of thrombin generation (±40% reduction of the ETP), whereas significantly less TG inhibition was detected in cirrhosis patients (Fig 4A+4C). In addition, prothrombin conversion was attenuated the most in healthy individuals (25%), and the least in CP-C patients (5%). The level of inhibition of TG and prothrombin conversion by TM addition decreases with the severity of the disease. Interestingly, TM decreases thrombin generation by reducing the total amount of prothrombin that is converted throughout the experiment, rather than decreasing the maximum rate of prothrombin conversion.

**Fig 5:**
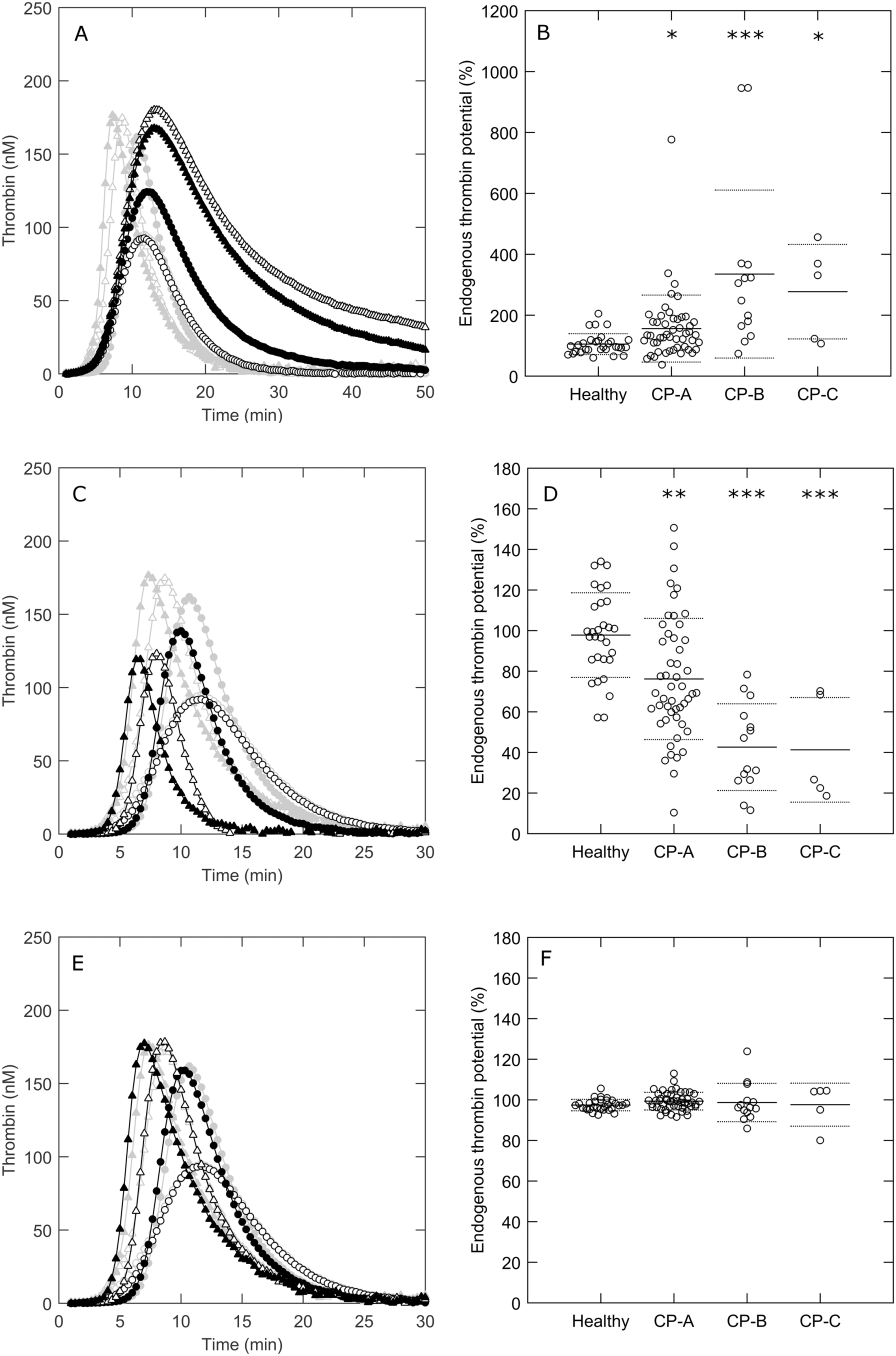
The contribution of changes in prothrombin conversion, antithrombin levels and a_2_-macroglobulin levels to the altered thrombin generation profile in cirrhosis patients. Prothrombin conversion (A-B), antithrombin levels (C-D) and α_2_-macroglobulin levels (E-F) were normalized *in silico* to the average level found in the healthy subjects group. Thrombin generation was simulated (black) in healthy subjects (○), Child-Pugh A patients (●), Child-Pugh B patients (Δ), Child-Pugh C patients (▲) and compared to the experimental values found in the same group of subjects (grey). The simulated and experimental TG curves in the healthy subject group are almost completely superimposed. The ratio of simulated and experimental ETP values were compared between patients and healthy subjects. *p<0.05, **p<0.01, ***p<0.001 compared to healthy subject values.

The thrombin generation profile of cirrhosis patients differs from that of healthy subjects because of (1) changes in prothrombin conversion, (2) antithrombin levels, and (3) α_2_-macroglobulin levels. We have performed *in silico* analysis to determine the individual contribution of these three changes to the overall process of TG (Fig 5). Firstly, we determined the effect of the reduction of prothrombin conversion in liver cirrhosis on TG by substituting prothrombin conversion curves from liver cirrhosis patients by healthy control prothrombin conversion curves (Fig 5A and 5B). If prothrombin conversion would be in the normal range in liver cirrhosis, this would cause the patient to be in a more procoagulant state, as thrombin generation would be markedly increased, especially in more severe cases of cirrhosis. Next, we determined the effect of acquired AT deficiency in cirrhosis patient by simulating TG curves as if patients had physiological AT levels (2.08 μM). Fig 5C and 5D shows that an increase in AT level in liver cirrhosis significantly decreases TG in patients, but not in healthy subjects. Finally, we tested whether an increase of α_2_M levels in cirrhosis patients has a protective function as it might counteract the pro-coagulant effects of an acquired AT deficiency, by predicting TG as if the α_2_M level of each patient was equal to the average healthy subject α_2_M (3.21 μM). Even though α_2_-macroglobulin plays a bigger role in thrombin inhibition in liver cirrhosis (Fig 4), this does not contribute significantly to thrombin generation in this context (Fig 5E and 5F).

**Fig 6:**
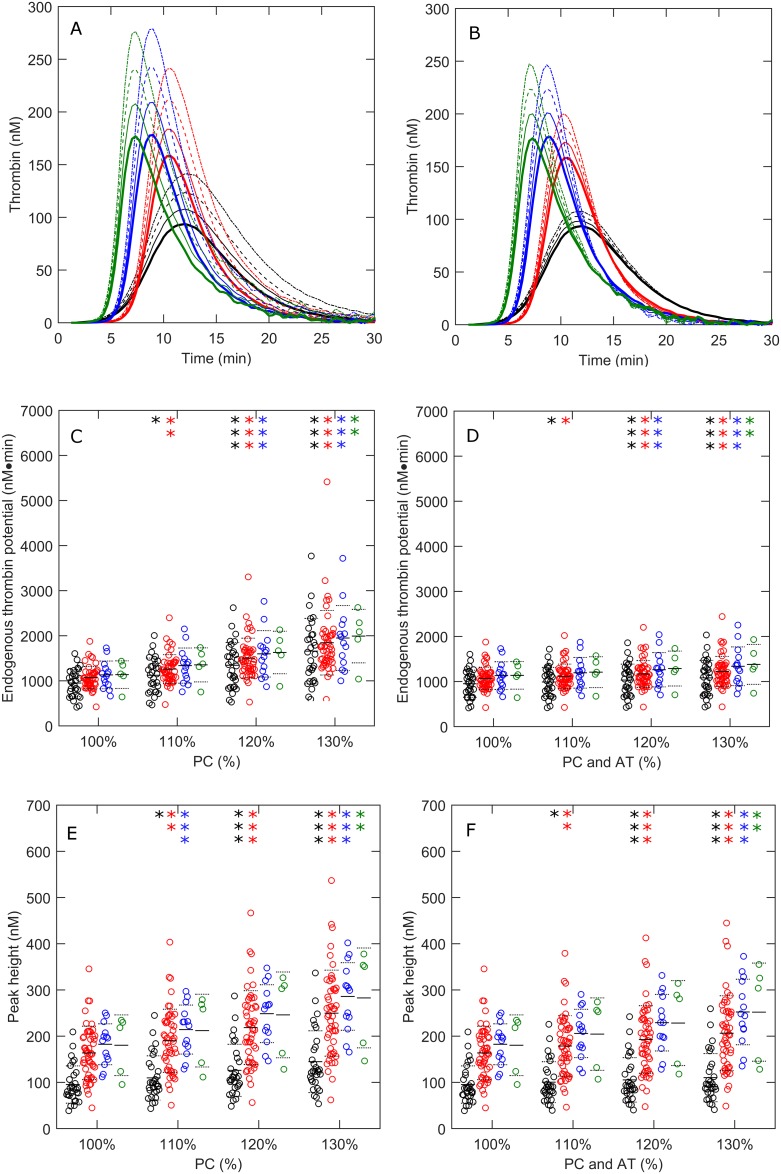
The *in silico* effect prothrombin complex concentrates with or without co-supplementation of antithrombin in cirrhosis. *In silico* experimentation was performed to predict the effect of prothrombin complex concentrate administration on thrombin generation in cirrhosis patients in the absence (A) or presence (B) of antithrombin supplementation. Prothrombin conversion curves were increased *in silico* to 110% (- -), 120% (- -), ad 130% (- ∙ -) with or without co-administration of 100%, 120%, or 130% AT. The effects of PCC with or without antithrombin were quantified by the ETP (C-D) and the peak height (E-F). Healthy subjects are shown in black, CP-A patients in red, CP-B patients in blue and CP-C patients in green. *p<0.05, **p<0.01, ***p<0.001 compared to healthy subject values.

Cirrhosis patients undergoing surgery often require transfusion products according to their clotting time (PT or aPTT). In Fig 6 we present an *in silico* prediction of thrombin generation after the administration of prothrombin complex concentrate by increasing the prothrombin conversion capacity of each subject to 110%, 120% and 130%. An increase of the prothrombin conversion capacity elevates ETP and peak height significantly (Fig 6C+6E) in healthy subjects and cirrhosis patients. In addition to PCC administration alone, we also predicted the effect of *in silico* administration of PCC in combination with antithrombin (Fig 6B). The combined administration of pro- and anticoagulant factors also increase the ETP and the peak height significantly, but to a lesser extent that PCC alone (Fig 6D+6F).

## Discussion

Our study confirms previous reports that AT and prothrombin levels are reduced in patients with liver cirrhosis [2–5,29] and that α_2_M levels are increased [30–33]. Thrombin generation peak height was found to be elevated at both low and high tissue factor concentrations, but the ETP was unchanged, as was reported by others [12,15,29,34–37]. Studies previously showed that liver cirrhosis patients do not suffer from unidirectional hemostatic problems (either bleeding or thrombosis) because their coagulation systems are rebalanced, as both their pro- and anticoagulant capacity decreases [1,6,38]. This explains the comparability of thrombin generation profiles between healthy subjects and cirrhosis patients. It is hard to imagine that the profound alteration of the coagulation factor profile in liver cirrhosis would not affect the processes of thrombin production and thrombin inactivation, which underlie the course of thrombin generation itself.

We showed that the total amount of prothrombin converted during TG is significantly reduced in liver cirrhosis patients, although the maximal velocity of prothrombin conversion is elevated in patients [20]. The reduction in prothrombin conversion is likely to be caused by a lower availability of prothrombin. The increased maximal rate of prothrombin conversion is dependent on the concentration or activity of the prothrombinase complex [39]. Potential mechanisms could be the reduction of antithrombin levels, causing reduced inhibition of FXa and thrombin or the attenuation of the inhibitory APC pathway. The latter is unlikely as the effect is also seen in TG measured in the absence of thrombomodulin which is required for the adequate function of the APC pathway during TG [40,41]. In addition, the reduction of AT levels (29%) in patients is reflected in a reduction of the thrombin decay capacity (30%). Even though α_2_M levels are increased in patients, α_2_M is incapable of restoring the thrombin decay capacity or thrombin generation itself in cirrhosis patients, but the percentage of thrombin that was inhibited by this secondary thrombin inhibitor was increased up to 3-fold compared to the values found in healthy subjects. Thus, in patients, less thrombin is formed, but it is formed faster and inactivated slower, causing an elevation of the thrombin generation peak height, but not the ETP. Nevertheless, we show that prothrombin conversion and thrombin inactivation are severely attenuated in cirrhosis, which provides experimental evidence that supports the hypothesis of rebalanced thrombin generation in cirrhosis. Similar to the prothrombin-antithrombin balance, we reckon with the possibility that the decrease of the factor V is compensated for by the decrease of the delimiting factors protein C and protein S. This is supported by the finding of thrombomodulin resistance in liver cirrhosis patients [35,36,42].

*In silico* experimentation pointed out that an increase of prothrombin conversion in liver cirrhosis patients to a physiological level will provoke a procoagulant state in CP-B and CP-C patients (ETP 300% of baseline on average), whereas the normalization of AT levels will cause a severe reduction of the ETP (half of all patients would have an ETP lower than 75% of normal). This indicates that indeed the coagulation system is rebalanced in liver cirrhosis patients, and that restoration of the prothrombin conversion capacity (e.g. based on a prolonged PT measurement) or thrombin decay capacity would result in an imbalanced coagulation system. Furthermore, we hypothesized that the increase in α_2_M level in liver cirrhosis could compensate for the AT deficiency to some extent. *In silico* experimentation shows that the increase of α_2_M levels in liver cirrhosis significantly improved the thrombin decay capacity, although it could not be restored to the level found in healthy subjects. However, the effect of elevated α_2_M levels in cirrhosis patients on TG itself was negligible and statistically insignificant. This is in line with the fact that AT is a much more potent thrombin inhibitor than α_2_M [43] and that a large increase in α_2_M level is needed to compensate a small loss of AT.

Even though a new hemostatic equilibrium seems to be established in liver cirrhosis, a higher incidence of bleeding and thrombotic episodes has been reported [6–11]. This may partly be due to local causes (e.g. varices), but also to a loss of buffer capacity, in the sense that small variations of a pro- and anticoagulant protein will result more easily in an unbalanced situation. Therefore, we can expect that patients react differently to anticoagulation treatment than healthy controls, as shown in recent literature [44]. The most obvious case is the decreased effectiveness of treatment with heparins, because heparin acts by increasing the AT activity [45–47]. Also the effects of direct FXa or thrombin inhibitors can be expected to show altered dynamics. Indeed, it was recently published by Potze et al. that the *in vitro* anticoagulant effect of rivaroxaban is decreased in patients with liver cirrhosis [48].

In addition, the transfusion of fresh-frozen plasma and other blood products based on a prolonged PT measurement has been under debate in liver cirrhosis [15,49,50]. Tripodi et al. showed that, although the administration of normal plasma shortened the PT, thrombin generation remained unchanged in liver cirrhosis patients [15].

We performed *in silico* experiments to investigate the effect of the transfusion of procoagulant factors alone or in combination with AT (to mimic prothrombin complex concentrates containing AT or fresh frozen plasma transfusion). We show that both transfusion protocols significantly increase TG, albeit procoagulant factors alone increases TG to levels associated with thrombosis, whereas procoagulant factors in combination with AT does not. This indicates that boosting the procoagulant system by administration of procoagulant factors based on an increased PT can even evoke a procoagulant state in liver cirrhosis patients. This indicates that pre-procedural correction of the PT with blood products may not be necessary, is potentially harmful [49,50], and stresses that the PT is not a global indicator of the coagulant state and reflects primarily procoagulant pathways. A global hemostasis test such as the thrombin generation measurement would be a better alternative to assess the hemostatic state of cirrhosis patients.

Computational simulation of thrombin generation can be profitably used for ‘what-if’ analysis. In complex diseases, such as liver cirrhosis, coagulation is altered at many points which have opposite effects on thrombin generation (e.g. increase of α_2_M and FVIII, and a decrease of FII and AT). This makes the integration of these changes and their net effect on the outcome hard to predict. Here we showed that, computational simulation can be used to determine the net outcome of the effect of a change of one or more factors in the system, which can be a physiological change or the administration of a drug [51]. This is especially useful to select optimal treatment option in liver cirrhosis patients.

This study has a few limitations, of which the first is that thrombin generation was not measured in platelet rich plasma and whole blood due to logistic difficulties. It would be interesting to study thrombin generation and especially prothrombin conversion in platelet rich plasma of liver cirrhosis patients in a future study, because platelet counts decrease with the severity of cirrhosis and the conversion of prothrombin into thrombin in platelet rich plasma is dependent on the procoagulant surface provided by activated platelets. In addition, patients receiving anticoagulant treatment were excluded because the aim of the study was to study the changes in the mechanism of thrombin generation in liver cirrhosis patients and not specifically the effect of anticoagulants.

Lastly, we currently only have a validated model for the interactions of thrombin with its inhibitors antithrombin and α_2_-macroglobulin [20]. An application of this model is that it can be used to split a thrombin generation curve into its underlying processes of prothrombin conversion and thrombin inactivation (the latter of which can be completely modeled). Unfortunately such a computational technique is not yet available for the APC system or the effects of FVIII, and therefore we could not yet investigate the role of the APC system or FVIII *in silico* in the current study.

In conclusion, we present experimental evidence that liver cirrhosis patients indeed have rebalanced thrombin generation. However, there are vast but concealed differences in the underlying mechanisms of prothrombin conversion and thrombin inactivation. This indicates that cirrhosis patients cannot be treated as hemostatically normal individuals. Caution is warranted in applying regular transfusion and anticoagulation protocols, especially if these procedures are based on a PT measurement, which overestimates the role of the procoagulant pathway in liver cirrhosis.

## Supporting information

S1 Fig Thrombin generation in cirrhosis patients and healthy subjects. (A) Mean thrombin generation curves in healthy subjects (○), Child-Pugh A patients (●), Child-Pugh B patients (Δ), and Child-Pugh C patients (▲) measured at 5 pM TF. (B) Lag time, (C) peak height, (D) endogenous thrombin potential, (E) time-to-peak and (F) velocity index were quantified from the TG curves. *p<0.05, **p<0.01, ***p<0.001 compared to healthy subject values.
(TIF)

S2 Fig Prothrombin conversion in cirrhosis patients and healthy subjects. (A) Mean prothrombin conversion curves in healthy subjects (○), Child-Pugh A patients (●), Child-Pugh B patients (Δ), and Child-Pugh C patients (▲) triggered with 5 pM TF. (B) Total prothrombin conversion, (C) maximal rate of prothrombin conversion, (D) thrombin-antithrombin formation, (E) thrombin-α_2_-macroglobulin formation and (F) the percentage of thrombin inhibited by α_2_-macroglobulin were quantified from the TG curves. *p<0.05, **p<0.01, ***p<0.001 compared to healthy subject values.
(TIF)
