# Supplementary figures and images for: Decreased prothrombin conversion and reduced thrombin inactivation explain rebalanced thrombin generation in liver cirrhosis
Source: PLoS One. 2017 May 4;12(5):e0177020. doi: 10.1371/journal.pone.0177020 (PMC5417641; doi:10.1371/journal.pone.0177020)

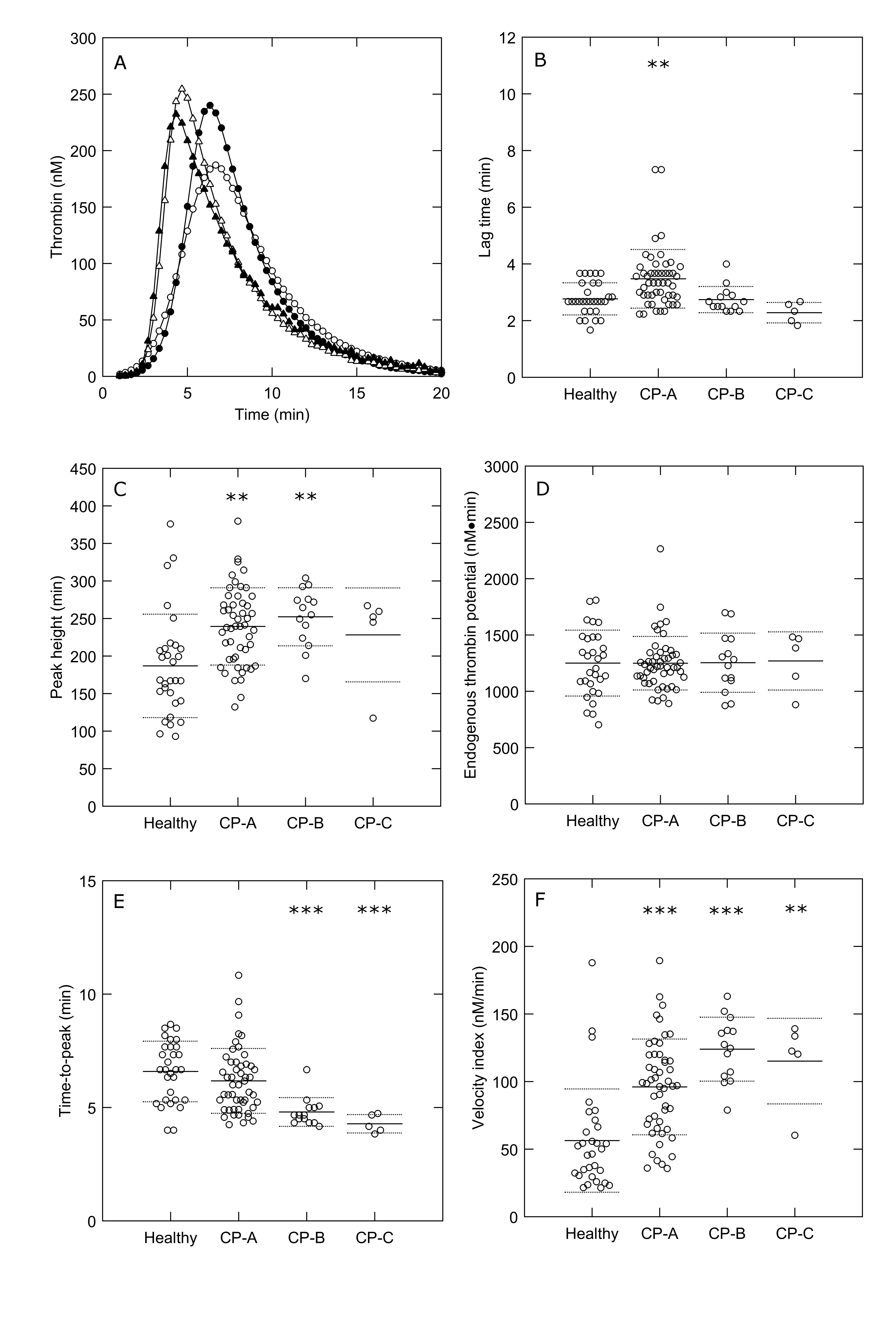

Supplement: S1 Fig — (A) Mean thrombin generation curves in healthy subjects (○), Child-Pugh A patients (●), Child-Pugh B patients (Δ), and Child-Pugh C patients (▲) measured at 5 pM TF. (B) Lag time, (C) peak height, (D) endogenous thrombin potential, (E) time-to-peak and (F) velocity index were quantified from the TG curves. *p<0.05, **p<0.01, ***p<0.001 compared to healthy subject values. (TIF) [file pone.0177020.s001.tif]

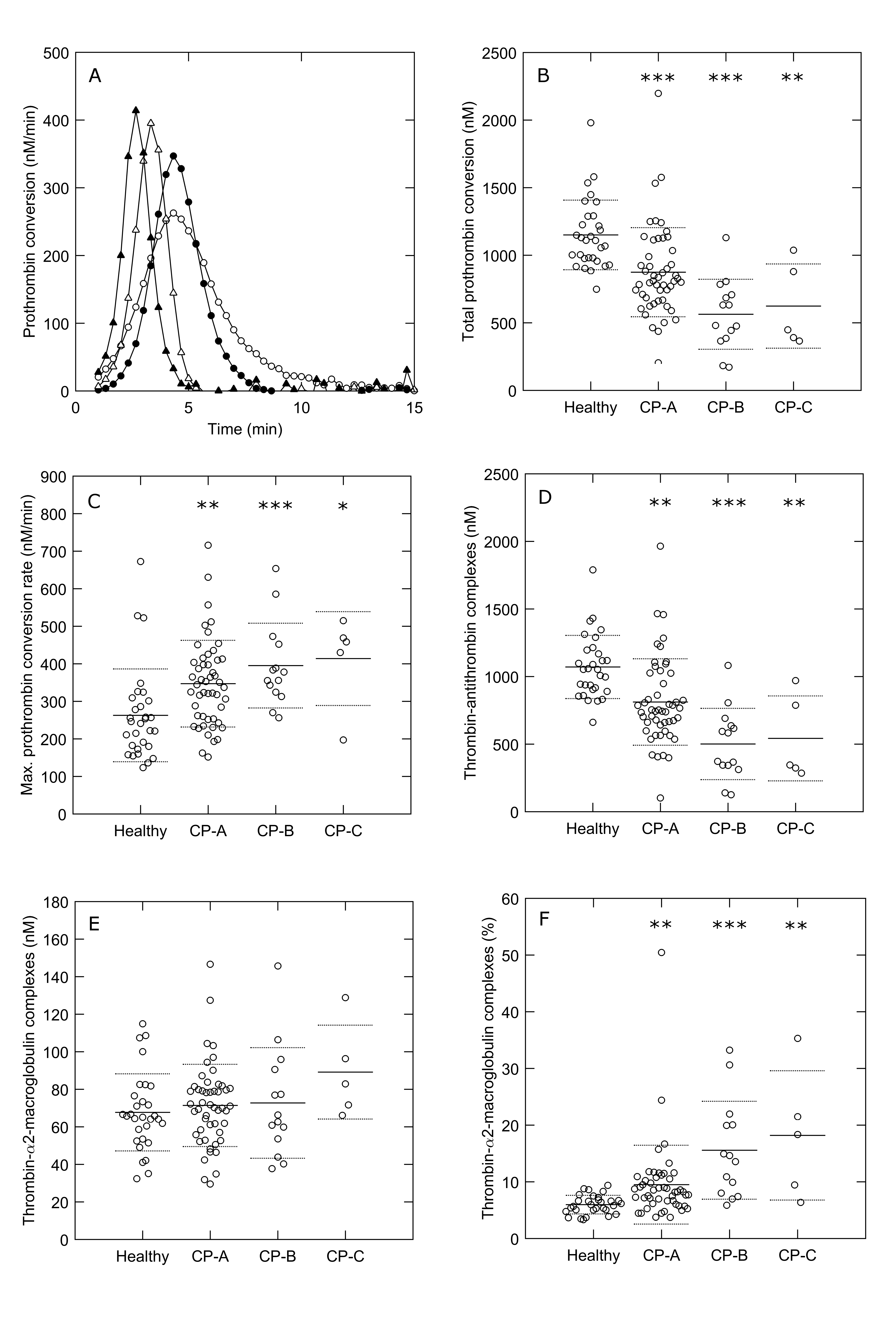

Supplement: S2 Fig — (A) Mean prothrombin conversion curves in healthy subjects (○), Child-Pugh A patients (●), Child-Pugh B patients (Δ), and Child-Pugh C patients (▲) triggered with 5 pM TF. (B) Total prothrombin conversion, (C) maximal rate of prothrombin conversion, (D) thrombin-antithrombin formation, (E) thrombin-α2-macroglobulin formation and (F) the percentage of thrombin inhibited by α2-macroglobulin were quantified from the TG curves. *p<0.05, **p<0.01, ***p<0.001 compared to healthy subject values. (TIF) [file pone.0177020.s002.tif]
